# Implementing Silicon Nanoribbon Field-Effect Transistors as Arrays for Multiple Ion Detection

**DOI:** 10.3390/bios6020021

**Published:** 2016-05-06

**Authors:** Ralph L. Stoop, Mathias Wipf, Steffen Müller, Kristine Bedner, Iain A. Wright, Colin J. Martin, Edwin C. Constable, Axel Fanget, Christian Schönenberger, Michel Calame

**Affiliations:** 1 Department of Physics, University of Basel, Basel 4056, Switzerland; mathias.wipf@gmail.com (M.W.); axel.fanget@unibas.ch (A.F.); Christian.Schoenenberger@unibas.ch (C.S.); michel.calame@unibas.ch (M.C.); 2 Department of Chemistry, University of Basel, Basel 4056, Switzerland; muelst@gmx.de (S.M.); iain.wright@durham.ac.uk (I.A.W.); colinmtn@gmail.com (C.J.M.); edwin.constable@unibas.ch (E.C.C.); 3 Laboratory for Micro- and Nanotechnology, Paul Scherrer Institute, Villigen 5232, Switzerland; KristineBedner@gmx.net; 4 Swiss Nanoscience Institute, University of Basel, Basel 4056, Switzerland

**Keywords:** chemical sensing, nanoribbons, sodium, fluoride, gold, ion-sensitive field-effect transistors, chemFETs

## Abstract

Ionic gradients play a crucial role in the physiology of the human body, ranging from metabolism in cells to muscle contractions or brain activities. To monitor these ions, inexpensive, label-free chemical sensing devices are needed. Field-effect transistors (FETs) based on silicon (Si) nanowires or nanoribbons (NRs) have a great potential as future biochemical sensors as they allow for the integration in microscopic devices at low production costs. Integrating NRs in dense arrays on a single chip expands the field of applications to implantable electrodes or multifunctional chemical sensing platforms. Ideally, such a platform is capable of detecting numerous species in a complex analyte. Here, we demonstrate the basis for simultaneous sodium and fluoride ion detection with a single sensor chip consisting of arrays of gold-coated SiNR FETs. A microfluidic system with individual channels allows modifying the NR surfaces with self-assembled monolayers of two types of ion receptors sensitive to sodium and fluoride ions. The functionalization procedure results in a differential setup having active fluoride- and sodium-sensitive NRs together with bare gold control NRs on the same chip. Comparing functionalized NRs with control NRs allows the compensation of non-specific contributions from changes in the background electrolyte concentration and reveals the response to the targeted species.

## 1. Introduction

Ions play a crucial physiological role for a large number of processes at the cellular level. The mis-regulation of their local concentration is suspected to be an indication or cause of various diseases including epilepsy or Alzheimer’s diseases [1,2]. Monitoring local ion gradients could improve the early detection of these diseases—A decisive advantage for the treatment [3,4]. Since the ionic regulation takes place at the intracellular and intercellular level, a meaningful measurement requires the sensing unit to be of the same length scale as the cells, *i.e.*, *μ*m-scale or smaller. This ultimately requires highly miniaturized sensing devices. Furthermore, physiological processes typically involve various different ionic species. The miniaturized sensing device must therefore also be capable of specifically detecting multiple ions in parallel to be used as future *in vivo* sensors. State-of-the-art chemical sensors are based on ion-selective electrodes (ISEs), which still require large volume analyte solutions not suitable for most medical applications. Additionally, their integration in microfluidic platforms for miniaturized chemical sensors remains a challenging, yet ongoing task and their potential for large-scale production is limited. On the contrary, systems based on field-effect transistors (FETs) have a great potential as cheap biochemical sensors. The possibility of integrating many sensors on a small area using well-established micro- and nanofabrication techniques makes these devices not only ideal candidates for *in vitro* point-of-care diagnostics but also as implanted devices for *in vivo* monitoring. However, the potential of FET arrays can only be fully exploited if different functionalities (e.g., multiple analyte detection) can be implemented on a single chip. In case of ion detection, this vision might lead to on-chip, spatially-resolved multiple ion sensing to locally monitor ionic gradients in small, compartmentalized units or in the extracellular matrix in the future [5]. In our previous work [6,7], we have demonstrated that arrays of gold-coated silicon (Si) nanowires or nanoribbons (NRs) can be used to detect a targeted ion. The gold layer allows anchoring ion receptors covalently at the sensor surface and minimizes the effect of pH as competing reaction due to its low pH sensitivity [7].

In this work, we expand our investigations of gold-coated Si NR arrays as multifunctional chemical sensors for the simultaneous detection of sodium and fluoride ions. Our approach includes two different ion receptors: a fluoride-sensitive transition metal complex and a sodium-sensitive crown ether. A microfluidic system based on polydimethylsiloxane (PDMS) is used to functionalize the gold surface of one NR group with self-assembled monolayers (SAMs) of fluoride-sensitive ligands and another NR group with SAMs of sodium-sensitive ligands. The functionalization leads to two types of active NRs: ${\mathrm{NR}}^{-}$ ($F^{-}$-sensitive) and ${\mathrm{NR}}^{+}$ (${\mathrm{Na}}^{+}$-sensitive). The remaining NRs (${\mathrm{NR}}^{c}$) are left untreated acting as a control. The procedure results in a differential setup having both active and control, bare gold-coated NRs on the same chip. This allows accounting for drift and reveals contributions of non-specific adsorption. The proposed functionalization procedure in combination with NR arrays is an important step towards a highly integrated sensing platform capable of multiplexing various chemical information into electrical signals.

## 2. Context and Basic Operation Principles

Operating FETs in a liquid environment has led to the ion-sensitive field-effect transistor (ISFET), a concept already introduced in the beginning of the 1970s [8]. These devices are currently being intensively studied at the nanoscale. In particular, silicon nanowire and silicon nanoribbon FETs have been used successfully for numerous sensing experiments such as pH sensing [9,10,11,12], chemical [6,13,14,15,16,17,18,19], and label-free biosensing [9,20,21,22,23,24,25].

The working principle of ISFETs is based on the gating effect induced by charged particles such as ions or proteins adsorbed at the sensor surface. To adsorb a certain analyte, the sensor surface needs to exhibit surface groups which interact with the targeted species, ideally with a high specificity. The reaction builds up a surface charge which results in a potential drop between the surface and the electrolyte called surface potential $\Psi_{0}$. Changes in $\Psi_{0}$ influence the charge carrier density of the underlying semiconducting channel. The change in $\Psi_{0}$ can be read out in the transistor characteristics as a shift of the transfer curve, here quantified by the threshold voltage $V_{th}$. For a p-type semiconductor operated in the accumulation regime as studied here, $\Delta V_{th}$ and $\Delta \Psi_{0}$ are directly connected via

(1) $$\Delta \Psi_{0}=-\Delta V_{th}$$

when the ISFET is used as a pH sensor, the gate dielectric, usually a thin oxide layer, is in direct contact with the analyte solution. Depending on the pH of the solution, a certain surface charge builds up due to protonation and deprotonation of the surface hydroxyl groups. High-k oxide surfaces such as ${\text{Al}}_{2}O_{3}$ or ${\text{HfO}}_{2}$ exhibit a high density of surface hydroxyl groups. Thanks to these materials, responses up to the Nernst limit (59.5 mV/pH at 300 K) have been demonstrated [9,10,11,12,26]. To specifically detect ions other than protons, the surface needs to be modified as we will further discuss in the Methods section.

## 3. Materials and Methods

### 3.1. Device Fabrication

SiNR ISFETs were fabricated by a top-down approach using electron-beam lithography on p-type silicon-on-insulator wafers (SOI, Soitec France, Grenoble, France) with a box oxide layer thickness of 145 nm. A detailed description can be found elsewhere [26]. The resulting NRs are 6μm in length, 85nm in height and of two different widths, 1μm and 25μm. The NR height of 85nm and the NR widths of 1μm and 25μm are larger than the optimal dimensions (thickness around 40nm and width around 100nm or smaller) found by numerical simulations [27,28]. However, the NR dimensions used in this study are a reasonable trade-off between low noise, high integration and easy fabrication as further discussed in our previous work [29]. Note that in this work, we use the term nanoribbon for the device structures although the actual lateral device dimensions are ≥1μm. This is justified by the fact that our top-down process allows fabricating structures with widths as small as 100nm as demonstrated in our previous work [26,29].

To ensure stable operation in liquid, the silicon channel is covered by 20nm of ${\text{Al}}_{2}O_{3}$ as gate oxide using atomic layer deposition (ALD). The good quality of the ALD oxide ensures low hysteresis and low leakage currents [30]. In addition, ${\text{Al}}_{2}O_{3}$ surfaces are highly sensitive to protons and responses up to the Nernstian maximum can be achieved. For specific ion detection, the high pH sensitivity of oxide surfaces leads to additional, undesired contributions to the measured signal as recently discussed in a previous work [7]. To minimize this influence of pH on the sensor signal, we coat the oxide surface with an additional gold layer of 20nm (with 5nm chromium as adhesion layer) by electron-beam evaporation. With the gold film, the pH response was shown to be around 30mV/pH in the range from pH 3 to pH 10, which is attributed to the formation of gold oxide. We estimate the amount of oxidized surface gold atoms to be around 1% [6]. The gold surface not only partially suppresses the response to pH but also allows using well-established thiol-based surface chemistry to functionalize the gold surface. This simplifies the functionalization procedure due to the possibility of single step monolayer formation. Furthermore, the gold layer does not affect the gate oxide capacitance, making it an ideal platform for surface functionalizations. The last step before functionalization includes wire-bonding into a chip carrier and epoxy sealing of the contacts (Epotek 353ND, Epoxy Technology). The final device consists of 48 nanoribbons arranged in four spatially separated arrays with a common drain contact. Each array of 12 nanoribbons is further separated in four pixels, each containing 3 nanoribbons. In each pixel, two nanoribbons have a width of 1μm and one a width of 25μm. For further details on the device layout, see Supplementary Materials.

### 3.2. Surface Functionalization

**Figure 1:**
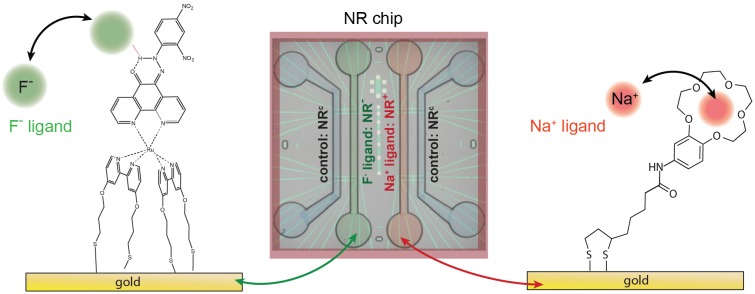
Schematics of the functionalization setup and molecular structure of the $F^{-}$ ligand (**Left**) and the ${\text{Na}}^{+}$ ligand (**Right**) immobilized on the gold surface; (**Middle**) schematics of the NR chip covered by microfluidic cell. Four channels are incorporated in our design, each containing 12 NRs (see Supplementary Materials for further details). The functionalization results in 24 functionalized NRs (12 ${\text{NR}}^{+}$, 12 ${\text{NR}}^{-}$) and 24 control NRs (${\text{NR}}^{c}$).

To achieve the parallel detection of multiple species with a single chip, the functionalization procedure must result in different surfaces, each specific to a certain target. We functionalize the gold surface of the SiNR FETs with self-assembled monolayers (SAMs) of two different ion receptors as illustrated in Figure 1. The first molecule ($F^{-}$ ligand) comprises a metal complex and a fluoride receptive phenathroline ligand which binds fluoride ions ($F^{-}$). The second molecule (${\text{Na}}^{+}$ ligand) consists of a 15-crown-5 crown ether structure attached to a dithiolane anchoring moiety. The structure has a high affinity towards sodium ions (${\text{Na}}^{+}$) and responses up to -44mV per decade in NaCl concentration have been achieved using gold-coated nanowires [6]. To functionalize the chip, we use PDMS microchannels. The channels were produced by pouring PDMS (Sylgard 184 Silicone elastomer, Dow Corning, Midland (Michigan), USA) onto SU-8 patterned Si wafers and curing at $60{\;}^{{}^{\circ}}C$ for 2 h. Four channels are incorporated in our design, each containing 12 NRs as depicted in Figure 1. The ion receptors were dissolved in methanol (≈1 mM). The sample was cleaned by UV/ozone and closed with the PDMS microchannel. Polytetrafluoroethylene (PTFE) tubes were used to connect the two active microchannels to a peristaltic pump (MCP, Ismatec) and the two solutions containing the ion receptors. SAMs were obtained by pumping the solutions through the channels with long stabilization times for 12 h. We functionalized the NRs in one channel with $F^{-}$ ligands (resulting in ${\text{NR}}^{-}$) and the NRs in another channel with ${\text{Na}}^{+}$ ligands (resulting in ${\text{NR}}^{+}$). The nanoribbons in the two remaining channels were used as a control (${\text{NR}}^{c}$) to monitor any changes in background electrolyte concentration and pH. This results in a differential setup having both active ${\text{NR}}^{-}$ and ${\text{NR}}^{+}$ and ${\text{NR}}^{c}$ on the same chip. After the functionalization, the active channels were flushed with methanol for 10min. Finally, the PDMS cell was removed and the samples were flushed with DI-water.

### 3.3. Buffer Solutions

Standard pH buffer solutions were used for the pH measurement (Titrisol, Merck, Darmstadt, Germany). NaF (ACS ≥99%, Sigma-Aldrich, St. Louis, MO, USA), NaCl (≥99.5%, Fluka (Sigma-Aldrich), St. Louis, MO, USA) and KCl (ACS 99.0%–100.5%, Sigma-Aldrich, St. Louis, MO, USA) were dissolved in deionized water (resistivity =18MΩcm) and buffered around pH7 with HEPES (≈4 mM, AppliChem, Darmstadt, Germany) and solution of KOH (≈1.5 mM, Merck, Darmstadt, Germany).

### 3.4. Measurement Setup

**Figure 2:**
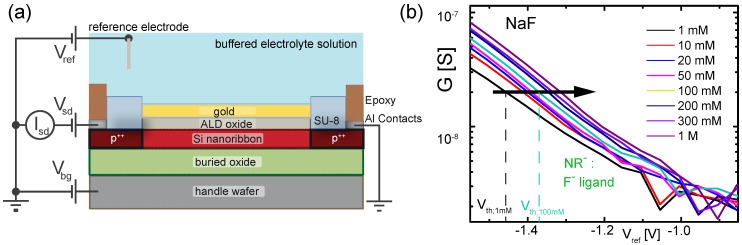
(**a**) measurement setup and device cross section. The working point of the transistor can be controlled by the liquid gate potential $V_{ref}$ applied to the reference electrode or by the backgate voltage $V_{bg}$ applied to the handle wafer. A constant source-drain voltage $V_{sd}=100\;\text{mV}$ is applied and the current through the channel $I_{sd}$ is measured; (**b**) conductance G *versus* liquid gate potential $V_{ref}$ of a 1μm-wide nanoribbon functionalized with $F^{-}$ ligands measured in buffered solutions with increasing NaF concentrations. The curves shift to the right with increasing concentration indicating adsorption of negatively charged species at the surface. The threshold voltage is determined as the value of $V_{ref}$ at a constant conductance value in the subthreshold as indicated by the black arrow.

In Figure 2a, the measurement setup is schematically depicted. A Keithley 2636a source meter (Keithley, Cleveland, OH, USA) is used to apply a constant source-drain voltage $V_{sd}$ of 100mV and to measure the source-drain current $I_{sd}$. A switching box (Keithley 3706, Keithley, Cleveland, OH, USA) is used to switch between different nanoribbons. The back-gate voltage $V_{bg}$ is applied to the handle wafer. $V_{bg}=0\;V$ for all measurements in this work. The liquid gate $V_{ref}$ is applied directly to the Ag/AgCl reference electrode (MI-401, Microelectrodes, Inc. Bedford, NH, USA) mounted on a polyetheretherketone (PEEK) flow cell with a total volume of ∼15μL which is pressed on the chip and sealed by an O-ring. Further details on the flow cell are given in the Supplementary Materials. Prior to a measurement series, the sample was stabilized in the buffer solution for ≈1 h. To determine the shift of the surface potential via $V_{th}$ the conductance of each NR was sequentially measured while sweeping the liquid gate potential. This results in a transfer curve for each NR measured in a specific analyte solution. Then, the solution was exchanged. After the solution exchange, the procedure was paused for a short stabilization time of 2min before the actual measurement was started. This procedure was repeated for all the solutions.

## 4. Results and Discussion

**Figure 3:**
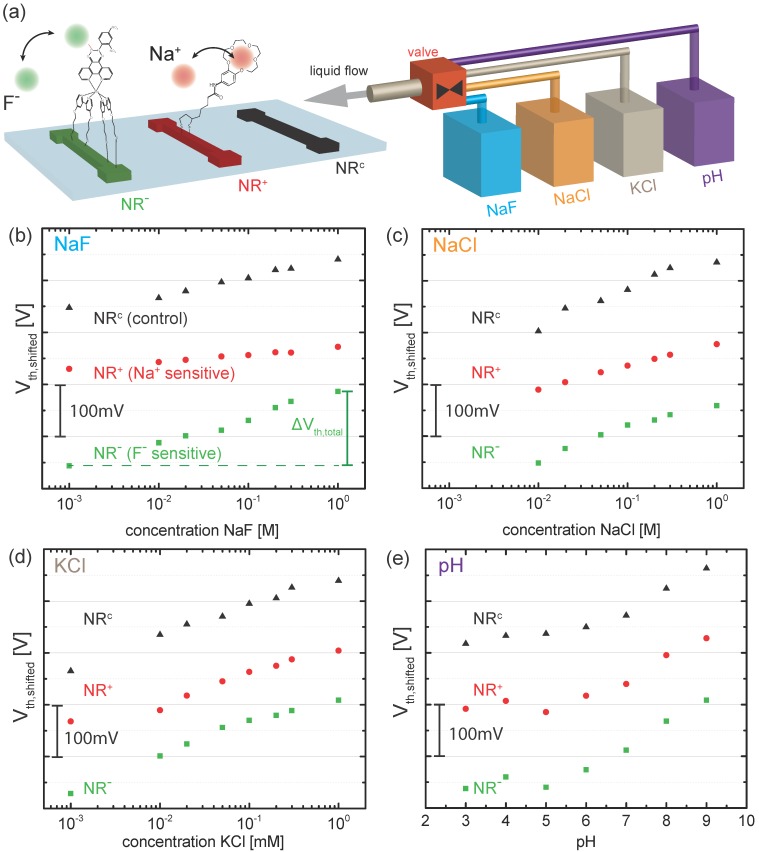
(**a**) schematics of the ion detection experiment. The response of a specific triplet of NRs consisting of an NR functionalized with SAMs of $F^{-}$ ligands (${\text{NR}}^{-}$, **green**), an NR functionalized with SAMs of ${\text{Na}}^{+}$ ligands (${\text{NR}}^{+}$, **red**) and an NR with bare gold surface (${\text{NR}}^{c}$, **black**) is measured in the presence of NaF (**blue**), NaCl (**orange**), KCl (**brown**) and pH (**violet**); (**b**–**e**) Experimental data (threshold voltage $V_{th}$ *versus* concentration) for (**b**) NaF, (**c**) NaCl, (**d**) KCl and (**e**) pH. Note that the experimental points of each NR was shifted along the vertical axis leading to $V_{th,shifted}$. Therefore, the absolute value of $V_{th,shifted}$ have been removed. The total change in threshold voltage $\Delta V_{th,total}$ is defined by the difference of $V_{th}\;\left(1\;M\right)-V_{th}\;\left(1\;\text{mM}\right)$ as indicated in (**b**).

In Figure 2b, we plot the transfer curves (conductance *G* *versus* liquid gate potential $V_{ref}$) for a gold-coated nanoribbon functionalized with a SAM of $F^{-}$ ligands (${\text{NR}}^{-}$) measured in buffered solutions with varying NaF concentration from 1mM to 1M. The curves shift to the right indicating the adsorption of negatively charged $F^{-}$ ions. To quantify the shift, we extract the threshold voltage $V_{th}$ for each transfer curve using our well-established method [6,7,12,30,31] by reading out the value of $V_{ref}$ at a constant conductance value of 20nS in the subthreshold regime of the transistor (black arrow in Figure 2b). In the following, we use $V_{th}$ to quantify the response of the different nanoribbons to changes in electrolyte concentration. In total, the response of a subset consisting of 14 out of 48 nanoribbons (4 ${\text{NR}}^{-}$, 4 ${\text{NR}}^{+}$ and 6 ${\text{NR}}^{c}$) was measured in order to minimize the measurement time. For the sake of clarity, we discuss here the results for a specific NR triplet consisting of one ${\text{NR}}^{-}$, one ${\text{NR}}^{+}$ and one ${\text{NR}}^{c}$ as depicted in Figure 3a. More information on the reproducibility and distribution of responses is given in the Supplementary Materials. The ribbons were chosen as such to represent functioning devices, showing a similar behavior in the control measurements in KCl and pH solutions as observed in previous measurements [6]. In the following, we compare the response of these three devices measured for increasing salt concentration (1mM to 1M) of NaF, NaCl and KCl and changing pH from pH 3 to pH 9 (Figure 3b–e). In particular, we investigate whether we can discriminate between sodium and fluoride ions by comparing the response of ${\text{NR}}^{-}$ and ${\text{NR}}^{+}$ with the control ${\text{NR}}^{c}$. Figure 3b shows the threshold voltages for the selected NR triplet in NaF solution. For the sake of readability, the experimental points from each NR were shifted along the vertical axis, leading to $V_{th;shifted}$. The original data is shown in the Supplementary Materials. Green squares correspond to $V_{th}$ for ${\text{NR}}^{-}$ shown in Figure 2b. The threshold voltage $V_{th}$ increases with salt concentration. We define the total change of the threshold voltage as $\Delta V_{th,total}=V_{th}\;\left(1\;M\right)-V_{th}\;\left(1\;\text{mM}\right)$. For ${\text{NR}}^{-}$, $\Delta V_{th,total}\approx 150\;\text{mV}$ as indicated in Figure 3b. Additionally, the threshold voltage of ${\text{NR}}^{c}$ (black triangles) and ${\text{NR}}^{+}$ (red circles) are shown. Note that ${\text{NR}}^{c}$ exhibits a response to changes in NaF concentration with $\Delta V_{th,total}\approx 100\;$mV. We attribute this response to the non-specific adsorption of fluoride ions at the bare gold surface, similarly to what we observed in our previous work for chloride ions [6,12]. Interestingly, ${\text{NR}}^{+}$ shows even a smaller $\Delta V_{th,total}\approx 50\;\text{mV}$ over the investigated concentration range. The observed behavior of the three different surfaces agrees well with the following picture: the largest response is observed for ${\text{NR}}^{-}$ due to the adsorption of fluoride ions at the SAM. The smaller response of ${\text{NR}}^{c}$ corresponds to the non-specific adsorption of fluoride ions. Therefore, we conclude that the response measured for ${\text{NR}}^{-}$ partially includes contributions from non-specific adsorption of fluoride ions at the gold surface. The smallest response is observed for ${\text{NR}}^{+}$ due to the additional adsorption of ${\text{Na}}^{+}$ ions in the crown ether, partially compensating the effect of non-specific fluoride adsorption. We repeated the measurement for the same set of NRs for increasing NaCl (Figure 3c) and KCl (Figure 3d) concentration. For both salts, ${\text{NR}}^{c}$ shows a response to changes in concentration due to the non-specific adsorption of chloride ions, in agreement with our previous work [6,7]. Furthermore, all three NRs exhibit a similar response to pH, as shown in Figure 3e, which is attributed to the presence of a low density of oxidized gold surface atoms [6].

**Figure 4:**
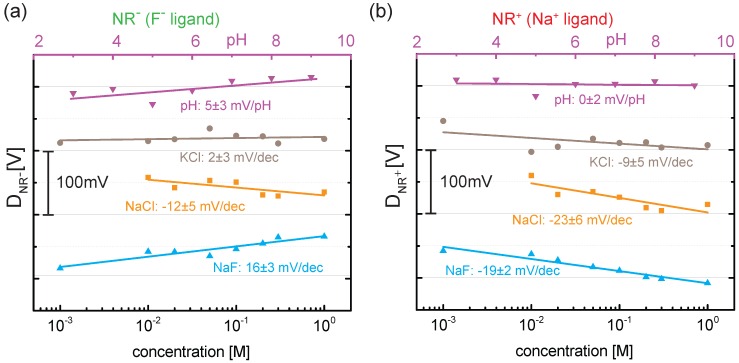
(**a**) differential response ($D_{{\text{NR}}^{-}}=V_{th;{\text{NR}}^{-}}-V_{th;{\text{NR}}^{c}}$) for ${\text{NR}}^{-}$ ($F^{-}$ ligand) and (**b**) differential response ($D_{{\text{NR}}^{+}}=V_{th;{\text{NR}}^{+}}-V_{th;{\text{NR}}^{c}}$) for ${\text{NR}}^{+}$ (${\text{Na}}^{+}$ ligand). In the case of NaF, the simultaneous detection of fluoride and sodium ions is achieved. Note that the pH has been changed by six orders of magnitudes (top horizontal axis) compared to three orders of magnitudes for the salt concentration (bottom horizontal axis).

To account for the non-specific anion adsorption at the gold surface, we follow the differential approach as introduced in our previous work [6,7]. Thereby, we subtract the threshold voltage of ${\text{NR}}^{c}$ ($V_{th,{\text{NR}}^{c}}$) from the two active NRs ($V_{th,{\text{NR}}^{+}}$ and $V_{th,{\text{NR}}^{-}}$) leading to the differential signal $D_{{\text{NR}}^{-}}=V_{th,{\text{NR}}^{-}}-V_{th,{\text{NR}}^{c}}$ for ${\text{NR}}^{-}$ and $D_{{\text{NR}}^{+}}=V_{th,{\text{NR}}^{+}}-V_{th,{\text{NR}}^{c}}$ for ${\text{NR}}^{+}$ as shown in Figure 4. It reveals the response of the two ligands (Figure 4a: $F^{-}$ ligand, Figure 4b: ${\text{Na}}^{+}$ ligand) and allows a quantitative comparison of the different surfaces. Negligible or weak responses to pH and changes in KCl concentration are observed for both ligands. This indicates that the functionalization does not influence the pH response and that neither potassium nor chloride ions bind to the two ligands. When changing NaCl and NaF concentration, however, a clearer differential response of ≈-20mV per decade (mV/dec) in salt concentration is observed for ${\text{NR}}^{+}$, which is due to the sensitivity of the ${\text{Na}}^{+}$ ligand to sodium. Note that the sign of the differential response indicates the adsorption of positively charged sodium ions. While ${\text{NR}}^{+}$ shows only a differential response when sodium ions are present, a similar behavior is expected from ${\text{NR}}^{-}$ for fluoride ions. However, due to the negatively charged fluoride ions, a positive differential response is predicted in this case. Indeed, we find for ${\text{NR}}^{-}$ a differential response of +16mV/dec in NaF due to the adsorption of $F^{-}$ at the SAM. Therefore, the simultaneous detection of sodium and fluoride ions in NaF is achieved. Finally, we also observe a differential response for ${\text{NR}}^{-}$ of -12mV/dec in NaCl which points towards some non-specific adsorption of sodium ions at the SAM. However, cation adsorption is not expected from the structure of the $F^{-}$ ligand and further measurements are needed to verify this finding.

We observe that the obtained responses are smaller than the maximum Nernst limit of 59.5mV/dec in ion concentration. This is a disadvantage compared to ISEs where thick membranes allow a Nernstian response over a large concentration range [32]. The sub-Nernstian response might be due to the relatively low ligand density at the sensor surface achieved. Using a very simplified site-binding model, we estimate the lower value of the density of $F^{-}$ ligands on ${\text{NR}}^{-}$ to be $N_{Ligand}^{F^{-}}\approx 5\times 10^{16}\;m^{-2}$ and the lower value of the density of ${\text{Na}}^{+}$ ligands on ${\text{NR}}^{+}$ to be $N_{Ligand}^{{\text{Na}}^{+}}\approx 7\times 10^{16}\;m^{-2}$. Note, these values are the lower estimates of the ligand density as discussed in the Supplementary Material. However, the studied ion receptors have not been optimized to achieve a high density on the surface, e.g., by minimizing their size. Comparing different NRs of the same surface reveals large variations in response (see Supplementary Materials). This indicates that our method of functionalization is prone to variations in final ligand density, which has a pronounced influence on the response, as described in our previous work [7]. The quality and the reproducibility of the SAM are therefore key elements for the further success of the presented approach. Although not demonstrated in this work, our approach could allow for the detection of mixed analyte solutions, where several types of anions and cations are present, given the response of individual NRs to the specific analytes is known. However, cross sensitivity limits the universality of this system and has to be taken into account. Nonetheless, our functionalization method results in an integrated sensing platform, and the simultaneous detection of sodium and fluoride ions is demonstrated.

## 5. Conclusions

In conclusion, we have demonstrated the simultaneous detection of sodium and fluoride ions using arrays of gold-coated SiNRs operated as ISFETs. Thanks to microfluidic channels incorporated in PDMS, we were able to functionalize individual parts of the sample with two different ion receptors sensitive to sodium and fluoride ions, while having control nanoribbons to monitor any changes in electrolyte concentration or pH. Our functionalization procedure results in a differential measurement setup having functionalized and control NRs on the same chip. Subtracting the background, the differential response reveals the response of the ligands. Using this setup, a differential response of ≈16mV/dec for $F^{-}$ and ≈-20mV/dec for ${\text{Na}}^{+}$ has been achieved. The sub-Nernstian responses are attributed to low ligand densities. Despite these difficulties, our differential approach is a very simple method to approximate the response of the specific adsorption of the targeted analyte on the functionalized NR. Upon proper calibration, it allows for quantifying the target analyte concentration.

Having different surfaces on the same chip expands the possibilities of classifying more complex solutions, even if perfect selectivity of the different surfaces is not given [33,34]. This is achieved by processing the acquired data of each NR using pattern recognition algorithms, in addition to calculating the differential response. Thanks to the ease of integration, arrays of gold-coated nanoribbon ISFETs offer a unique platform for point-of-care diagnostics.

## Supplementary Materials

The following are available online at www.mdpi.com/2079-6374/6/2/21/s1, Figure S1: Device Layout, Figure S2: Fluidic Setup, Figure S3: Raw data and detailed information on the ion receptors synthesis.

## Abbreviations

The following abbreviations are used in this manuscript:

FETs: field-effect transistor
Si: silicon
NRs: nanoribbons
PDMS: polydimethylsiloxane
ISFET: ion-sensitive field-effect transistor
ALD: atomic layer deposition
PTFE: polytetrafluoroethylene
