# Supplementary material for: Implementing Silicon Nanoribbon Field-Effect Transistors as Arrays for Multiple Ion Detection
Source: Biosensors (Basel). 2016 May 6;6(2):21. doi: 10.3390/bios6020021 (PMC4931481; doi:10.3390/bios6020021)
Supplement: Supplementary file 1 [file biosensors-06-00021-s001.pdf]

Supplementary Materials: Implementing Silicon Nanoribbon Field-Effect Transistors as Arrays for Multiple Ion Detection

Ralph L. Stoop, Mathias Wipf, Steffen Müller, Kristine Bedner, Iain A. Wright, Colin J. Martin, Edwin C. Constable, Axel Fanget, Christian Schönenberger and Michel Calame

1. Device Layout

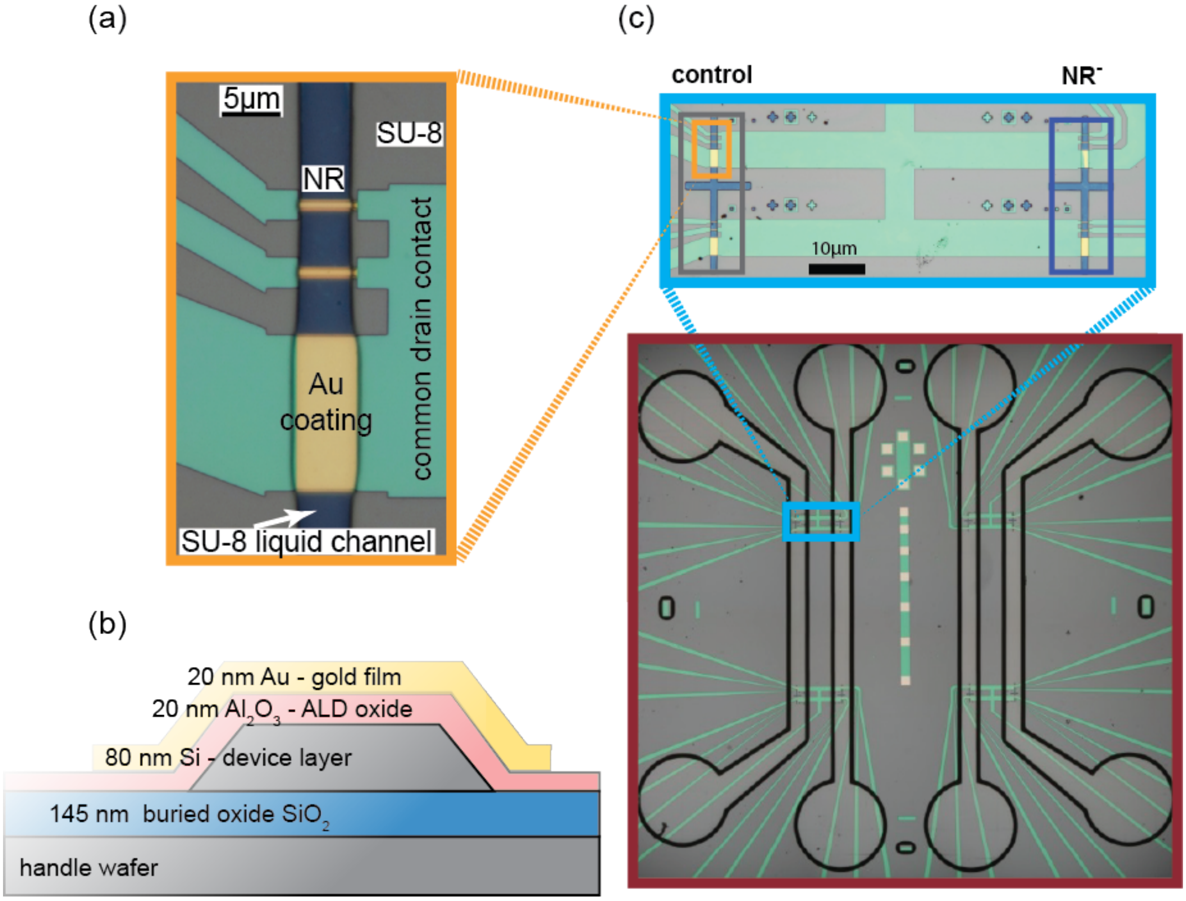

Figure S1. Device layout. (**a**) optical microscopy of a pixel consisting of two 1 μm-wide nanoribbons and a 25 μm-wide nanoribbon; (**b**) schematic of the nanoribbon cross section showing a 20 nm thick gold layer deposited on the Al_2O_3 layer. The thickness of the Si device layer is 80 nm; (**c**) large picture: Optical micrograph of the sample covered with a four channel polydimethylsiloxane (PDMS) microfluidic cell. Round regions denote in- and outlets for the tubing. Forty-eight nanoribbons (NRs) are arranged in four spatially separated arrays (**bottom**) consisting of 12 NRs (**top**). Each microfluidic channel contains 12 NRs. Small picture: Zoomed micrograph of the upper left NR array containing 12 NRs.

Fluidic Setup

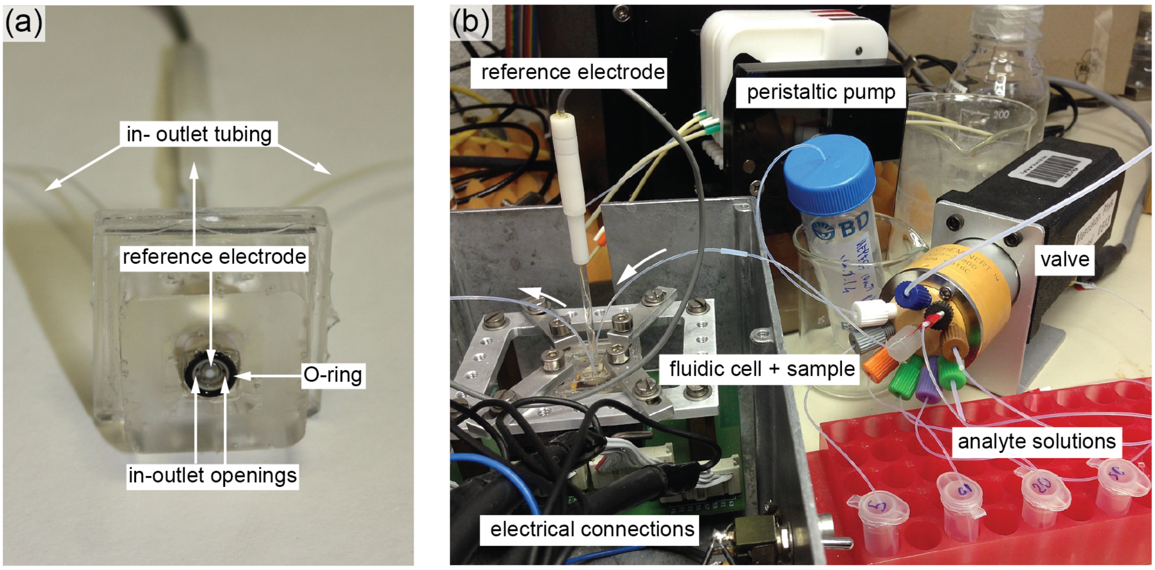

Figure S2. Fluidic setup. (**a**) measurement cell with the reference electrode mounted in the middle of the fluidic chamber; (**b**) liquid setup. A peristaltic pump is used to pull the analyte solutions through the valve to the fluidic cell.

2. Reproducibility

In total, the response of a subset consisting of 14 out of 48 nanoribbons (4 NR^{-}, 4 NR^{+} and 6 NR^{c}) was measured in order to minimize the measurement time as the source-drain current readout is done sequentially for each NR. The number of measured NRs does not yet allow a meaningful statistical analysis regarding average response and standard deviation. We did find variations in the response which we attribute to a degraded surface quality for this particular NR chip (Figure S3). The sample has been exposed to numerous harsh surface treatments during previous surface functionalizations and measurements. Although gold is commonly assumed to be a stable, inert material, surface degradation cannot be excluded. The result is an alteration of the gold film which leads to the observed variability in the response. However, during the reported measurements, no significant device degradation is observed. Therefore, we assume that the surface properties of individual NRs remains stable for the reported measurements. For NR^{-} and NR^{+}, the surface functionalization leads to additional variability among the ribbons. Although all NRs of a specific group were in contact with the same solution during functionalization, a certain spreading in the resulting densities of the SAMs is expected when using our proposed microchannel functionalization method. In summary, the reproducibility of the transistors must be guaranteed for the further success of this platform. This also includes the reproducibility of the SAM deposition which is not yet applicable in an industrial process. Despite the observed spreading in the response, we achieved a meaningful response of 16 mV/dec for fluoride and −20 mV/dec for sodium.

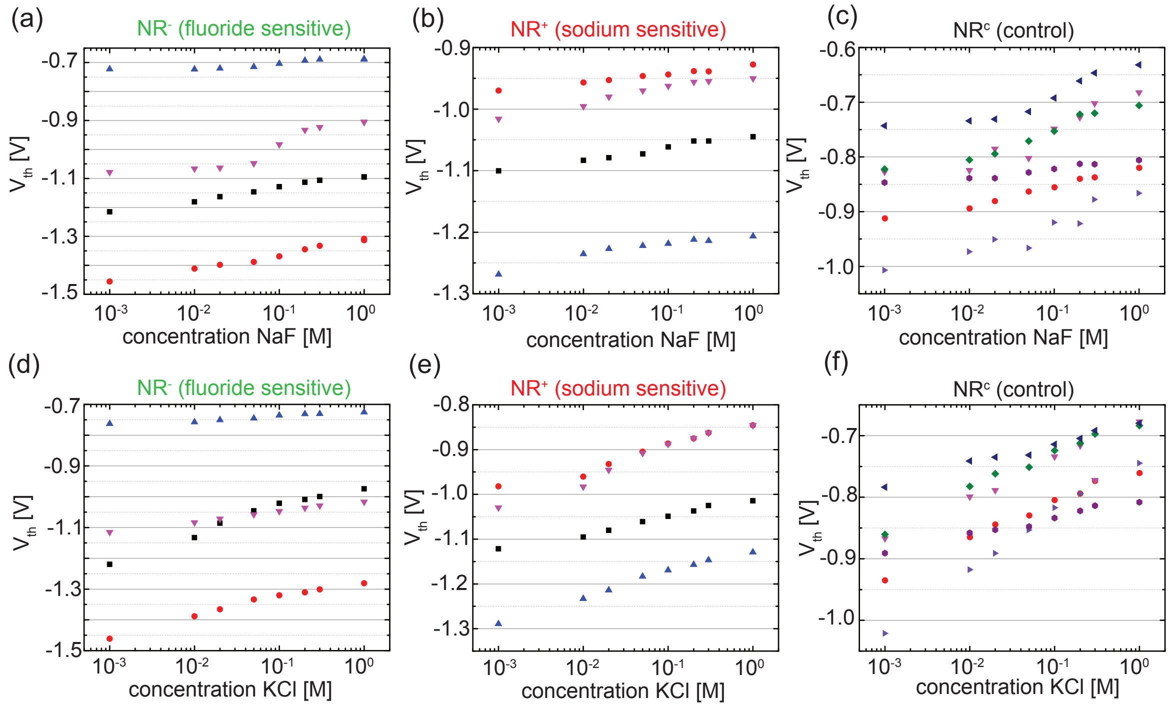

Figure S3. Raw data. V_{th} *versus* concentration of NaF (**a**–**c**) and KCl (**d**–**f**) for the fluoride-sensitive NR^- (**a**,**c**), sodium-sensitive NR^+ (**b**,**e**) and NR^c (**c**,**f**). V_{th} values of NRs shown in the manuscript are indicated by the red circles.

3. Estimation of the Ligand Densities

As described in the manuscript, we estimate the density of F^- ligands on NR^- to be $N^{F^-}_{Ligand} \approx 5 \times 10^{16}\,\mathrm{m}^{-2}$ and the density of Na^+ ligands on NR^+ to be $N^{Na^+}_{Ligand} \approx 7 \times 10^{16}\,\mathrm{m}^{-2}$. These estimations are based on an extended site-binding model as discussed in our previous work [1]. In short, the model assumes that the change in surface potential $\Delta\Psi_0$ due to ion adsorption of total charge Q is given by

$$\Delta\Psi_0 = \frac{Q}{C_{dl}} = \frac{\sigma}{C^{\square}_{dl}} \tag{1}$$

with C_{dl} the double layer capacitance at the sensor/electrolyte interface. $\sigma = Q/A$ is the charge density at the surface (with A the NR surface area) and $C^{\square}_{dl} = C_{dl}/A$ is the double layer capacitance per unit area. Q depends on the binding affinity of the ligand-ion system, the ligand density N_{Ligand} and the concentration of the ion as discussed in detail in our previous work [1]. The differential response shown in Figure 4 of the manuscript indicates how much the surface potential of the active NR changes compared to the control NR due to the additional adsorption of ions at the ligands of the active NR. Over the total investigated concentration range from 1 mM to 1 M, the differential response of NR^- to NaF (16 mV/dec) leads to a total shift of $\Delta^{NR^-}_{total}\Psi_0 \approx 50\,\mathrm{mV}$. Correspondingly, the differential response of NR^+ to NaCl (23 mV/dec) leads to a total shift of $\Delta^{NR^+}_{total}\Psi_0 \approx 70\,\mathrm{mV}$. From the total shift in surface potential of the two active NRs, we can calculate the corresponding charge Q or surface charge density σ by assuming a constant double layer capacitance of $C^{\square}_{dl} = 0.16\,\mathrm{Fm}^{-2}$. This leads to $\sigma_{NR^-} = \Delta^{NR^-}_{total}\Psi_0 \cdot C^{\square}_{dl} = 0.008\,\mathrm{Cm}^{-2}$ and $\sigma_{NR^+} = \Delta^{NR^+}_{total}\Psi_0 \cdot C^{\square}_{dl} = 0.0112\,\mathrm{Cm}^{-2}$. Note, σ consists of all ions adsorbed at the ligand monolayer. We further assume that each adsorbed ion is binding to exactly one ligand. In this case, it is evident that the actual ligand density has to be similar or larger than the calculated surface charge density divided by the elementary charge e

$$N_{Ligand} \geq \frac{\sigma}{e} \tag{2}$$

Therefore, the lower estimate of N_{Ligand} is given by $N_{Ligand} = \sigma/e$. Applied to the densities calculated for the active NRs, we obtain $N^{F^-}_{Ligand} = \sigma_{NR^-}/e = 5 \times 10^{16} m^{-2}$ for NR^- (F^- ligand) and $N^{Na^+}_{Ligand} = \sigma_{NR^+}/e = 7 \times 10^{16} m^{-2}$ for NR^+ (Na^+ ligand). The lower estimate of N_{Ligand} denotes the ligand density if the response observed in Figure 4 was due to full saturation of the sensor surface. In other words, the surface changes from completely unoccupied at 1 mM to fully saturated (each ligand is binding one ion) at 1 M. The actual ligand density might be considerably larger than the lower estimation, in the range of $0.8 \times 10^{17} m^{-2} - 4 \times 10^{17} m^{-2}$ [1]. Since, in our case, the ligand is much larger than the analyte, the ligand density is mainly limited by the ligand size and its surface arrangement properties. We do believe that further optimization of the ligand density is a key point for the future success of the platform.

4. Ligand Synthesis

4.1. General Experimental

Synthesis: All reagents and dry solvents were sourced commercially and used without further purification. Melting points were recorded using an A. Krüss Optronic M5000 (A. Krüss, Hamburg, Germany) and are uncorrected. ^1H and ^{13}C NMR spectra were recorded on a Bruker Avance III-500 (Bruker, Billerica, MA, USA); chemical shifts were referenced to residual solvent peaks with respect to δ(TMS) = 0 ppm. Electron impact (EI) mass spectra were recorded on a Finnigan MAT 95Q instrument (ThermoFisher Scientific, Waltham, MA, USA).

4.2. F^- Ligand Synthesis

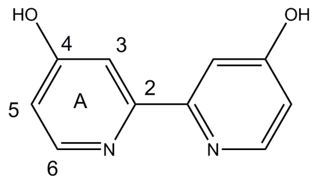

[2,2′-Bipyridine]-4,4′-diol (**OH-bpy**)

4,4′-Dimethoxy-2,2′-bipyridine (1.51 g, 7.0 mmol, 1.0 eq.) was dissolved in acetic acid (80 mL) and HBr (48 wt% sol. in water, 7.97 mL, 70.0 mmol, 10 eq.) was added. After refluxing for 24 h and cooling to room temperature, the formed precipitate was filtered off and dissolved in water. Neutralization of the solution with aqueous ammonia yielded precipitate which was filtered off, washed with water and dried. **OH-bpy** was obtained as a colorless solid (1.07 g, 5.68 mmol, 81 %).

^{1}**H-NMR** (250 MHz, D_2O + NaOH) δ/ppm: 8.04 (d, J = 6.3 Hz, 2H, H^{A6}), 6.99 (d, J = 2.5 Hz, 2H, H^{A3}), 6.58 (dd, J = 6.3, 2.5 Hz, 2H, H^{A5}). The ^{1}H-NMR spectroscopic data are in accordance with the literature [2].

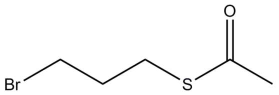

S-(3-Bromopropyl) ethanethioate (**SC2**)

Potassium thioacetate (2.1 g, 18 mmol, 1.0 eq.) and 1,3-dibromopropane (2.02 ml, 19.8 mmol, 1.1 eq.) were refluxed in THF (100 ml) for 2.5 h. After cooling, the mixture was stirred at room temperature for 3 h followed by removal of the solvent under reduced pressure. The residue was dissolved in CH_2Cl_2, filtered over celite and the solvent was removed *in vacuo*. **SC2** was obtained after distillation (65°C at $1 \cdot 10^{-1}$ mbar) as colorless oil (1.73 g, 8.78 mmol, 48%).

^{1}**H-NMR** (400 MHz, $CDCl_3$) δ/ppm: 3.44 (t, J = 6.5 Hz, 2H), 3.00 (t, J = 7.0 Hz, 2H), 2.33 (s, 3H, Me), 2.11 (m, 2H). The ^{1}H-NMR spectroscopic data are in accordance with the literature [3].

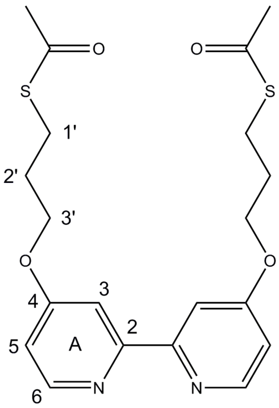

S,S′-(([2,2′-Bipyridine]-4,4′-diylbis(oxy))bis(propane-3,1-diyl)) diethanethioate (**L4-SAc**)

OH-bpy (226 mg, 1.2 mmol, 1.0 eq.) and K_2CO_3 (1.0 g, 7.24 mmol, 6.0 eq.) were added to a solution of **SC2** (500 mg, 2.54 mmol, 2.1 eq.) in dimethylformamide (DMF) (15 mL) and the reaction mixture was stirred for 6 h at 80 °C. After cooling and removal of the solvent, the residue was suspended in water and extracted three times with CH_2Cl_2. The combined organic fractions were dried over $MgSO_4$ and the solvent was removed. The crude product was purified by column chromatography (SiO_2, cyclohexane/ethyl acetate 1:6, Rf = 0.2). **L4-SAc** was isolated as a colorless solid (0.43 g, 1.02 mmol, 85%).

^{1}**H-NMR** (400 MHz, $CDCl_3$) δ/ppm: 8.46 (d, J = 5.7 Hz, 2H, H^{A6}), 7.95 (d, J = 2.5 Hz, 2H, H^{A3}), 6.83 (dd, J = 5.7, 2.6 Hz, 2H, H^{A5}), 4.18 (t, J = 6.0 Hz, 4H, $H^{3'}$), 3.07 (t, J = 7.1 Hz, 4H, $H^{1'}$), 2.34 (s, 6H, H^{Me}), 2.11 (m, 4H, $H^{2'}$). ^{13}**C-NMR** (126 MHz, $CDCl_3$) δ/ppm: 195.8 ($C^{C=O}$, 166.0 (C^{A4}), 158.0 (C^{A2}), 150.3 (C^{A6}), 111.4 (C^{A5}), 106.8 (C^{A3}), 66.3 ($C^{3'}$), 30.8 (C^{Me}), 29.1 ($C^{2'}$), 25.9 ($C^{1'}$). **MP**: 134 °C. **IR** (solid, ν/cm^{-1}): 509 (m), 537 (m), 577 (m), 625 (s), 753 (m), 827 (s), 857 (m), 928 (m), 953 (m), 987 (m), 1026 (m), 1065 (m), 1105 (m), 1134 (s), 1181 (m), 1223 (m), 1243 (s), 1294 (s), 1348 (m), 1384 (m), 1408 (m), 1438 (m), 1454 (m), 1507 (m), 1538 (m), 1560 (s), 1581 (s), 1630 (m), 1687 (s), 2930 (w). **MS** (ESI, m/z): 421.2 $[M+H]^+$ (calc. 421.1). **EA**: Found C 57.19%, H 5.83%, N 6.55%, $C_{20}H_{24}N_2O_4S_2$ requires C 57.12%, H 5.75%, N 6.66%.

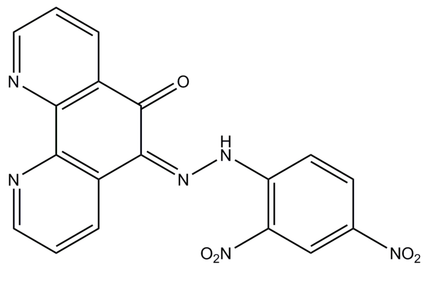

6-(2-(2,4-Dinitrophenyl)hydrazono)-1,10-phenanthrolin-5-one (**L5**)

The precursor 1,10- phenanthroline-5,6-dione (phen-dione) was synthesized following the procedure reported by Paw and Eisenberg [4]. Phen-dione (1.0 g, 4.76 mmol, 1.0 eq.) was suspended in EtOH (15 mL) and concentrated H_2SO_4 (3 mL) and added to a suspension of 2,4-dinitrophenyl hydrazine (1.62 g, 5.71 mmol, 1.2 eq.) in EtOH (15 ml) and concentrated H_2SO_4 (2 mL). The mixture was heated to reflux overnight. The formed orange precipitate was filtered off and washed with 5% aqueous $NaHCO_3$-solution to remove residual acid, then washed with water. The solid was stirred as a suspension in hot EtOH/acetone to remove precursors. After filtering and drying, the product **L5**

was obtained as a bright orange solid (1.62 g, 4.1 mmol, 87 %).

^{1}**H-NMR** (250 MHz, TFA-d) δ/ppm: 9.62 (dd, J = 8.4, 1.2 Hz, 1H), 9.40 (m, 2H), 9.25 (dd, J = 4.8, 1.5 Hz, 1H), 9.13 (m, 2H), 8.78 (d, J = 1.2 Hz, 2H), 8.33 (dd, J = 8.4, 5.6 Hz, 1H), 8.09 (dd, J = 8.1, 4.9 Hz, 1H).

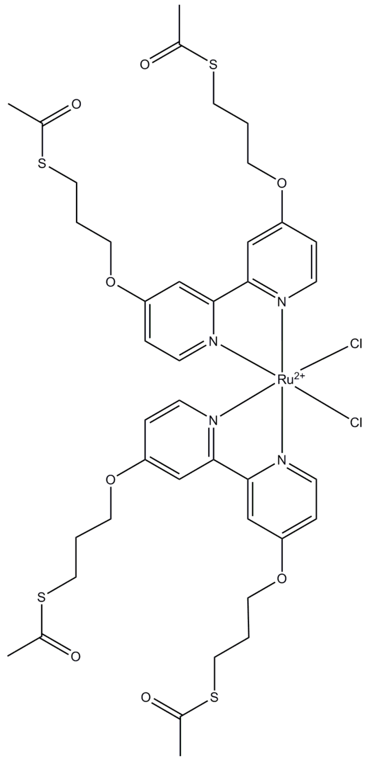

Ru(L4-SAc)$_2$Cl$_2$

A microwave vial was charged with RuCl$_2$(cod) (60 mg, 214 μmol, 1.0 eq.), **L4-SAc** (180 mg, 428 μmol, 2.0 eq.) and DMF (15 mL) and heated in a microwave reactor for 1 h at 100 °C. The solvent was removed and **Ru(L4-SAc)$_2$Cl$_2$** was obtained as a dark red solid without further purification. (215 mg, quant. yield).

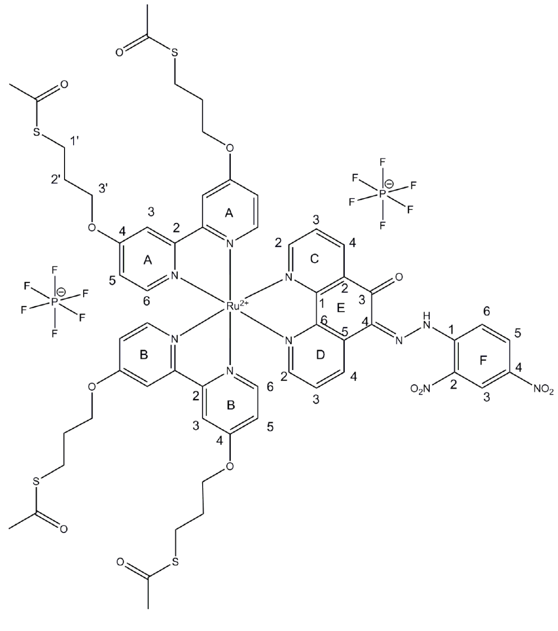

F^- Ligand

A mixture of **Ru(L4-SAc)2Cl$_2$** (100 mg, 99 μmol,1.0 eq.) and **L5** (46 mg, 118 μmol, 1.2 eq.) in MeOH (14 mL) was heated in a microwave reactor at 115 °C for 1.5 h. The resulting solution was poured into water and aqueous NH_4PF_6-solution was added. The formed precipitate was filtered over celite, washed with water and diethyl ether and dried in an airstream. The solid was dissolved in acetonitrile, the solvent was removed, and the red solid was dissolved in acetone (3 mL) and precipitated in petrol ether. Further purification was performed by recrystallization from EtOH. F^- ligand was obtained as a red solid (90 mg, 55 μM, 56 %).

^{1}**H-NMR** (500 MHz, CD_3CN) δ/ppm: 9.10 (d, J = 2.5 Hz, 1H, H^{F3}), 8.87 (dd, J = 8.4, 1.2 Hz, 1H, H^{D4}), 8.76 (dd, J = 8.0, 1.4 Hz, 1H, H^{C4}), 8.69 (d, J = 9.6 Hz, 1H, H^{F6}), 8.59 (dd, J = 9.3, 2.6 Hz, 1H, H^{F5}), 8.14 (dd, J = 5.4, 1.4 Hz, 1H, H^{C2}), 7.96 (d, J = 2.6 Hz, 4H, $H^{A3;B3}$), 7.93 (m, 1H, H^{D2}), 7.65 (dd, J = 8.0, 5.5 Hz, 1H, H^{C3}), 7.61 (m, 1H, H^{D3}), 7.51 (d, J = 6.5 Hz, 4H, $H^{A6;B6}$), 6.91 (m, 4H, $H^{A5;B5}$), 4.24 (t, J = 6.1 Hz, 8H, $H^{3'}$), 3.02 (t, J = 7.1 Hz, 8H, $H^{1'}$), 2.30 (s, 12H, H^{Me}), 2.08 (m, 8H, $H^{2'}$). ^{13}**C-NMR** (126 MHz, CD_3CN) δ/ppm: 196.3 ($C^{C=O}$), 179.0 (C^{E3}), 166.9 ($C^{A4,B4}$), 159.4 ($C^{A2,B2}$), 157.4 (C^{C2}), 157.0 (C^{E1}), 153.1 ($C^{A6,B6}$), 152.9 (C^{D2}) 149.8 (C^{E6}), 144.2 (C^{F4}), 143.5 (C^{F1}), 135.9 (C^{F2}), 135.5 (C^{C4}), 132.7 (C^{E5}), 131.8 (C^{E4}), 131.1 (C^{E2}), 131.0 (C^{F5}), 128.5 (C^{D3}) 128.1 (C^{C3}), 123.4 (C^{F3}), 119.9 (C^{F6}), 115.0 ($C^{A5,B5}$), 112.0 ($C^{A3,B3}$), 68.9 ($C^{3'}$), 30.8 (C^{Me}), 29.6 ($C^{2'}$), 26.0 ($C^{1'}$). **IR** (solid, ν/cm^{1}): 557 (m), 585 (s), 613 (s), 825 (s), 955 (m), 1028 (s), 1131 (m), 1211 (m), 1333 (m), 1438 (m), 1484 (m), 1608 (s), 1682 (m), 1973 (w), 2928 (w), 3235 (w). **MS** (ESI, m/z): 666.1 $[M\text{-}2PF_6]^{2+}$ (calc. 666.1), (MALDI-TOF, m/z) 1332.2 $[M\text{-}2PF_6]^+$ (calc. 1332.2). **EA**: Found C 42.60 %, H 4.13 %, N 7.03 %, $C_{58}H_{58}F_{12}N_{10}O_{13}P_2RuS_4 \cdot 3H_2O \cdot 4EtOH$ requires C 42.60 %, H 4.77 %, N 7.53 %

4.3. Na^+ Ligand Synthesis

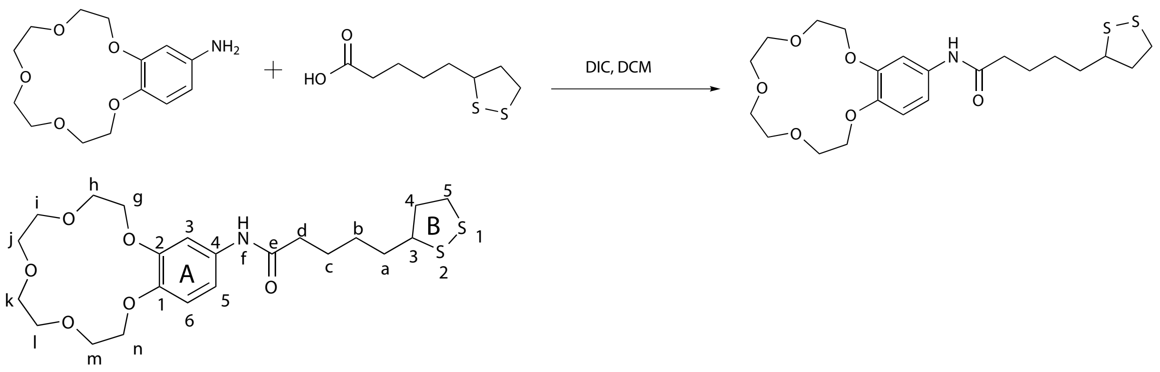

Figure S4. Reprinted with permission from [5]. Copyright 2013 American Chemical Society.

The Na^+ ligand was synthesized as described recently [5]. Briefly, 5-aminobenzo-15-crown-5 (1.06 g, 3.73 mmol), racemic lipoic acid (1.00 g, 4.85 mmol), and N,N′-diisopropyl carbodiimide (0.66 g, 5.22 mmol) were dissolved in anhydrous dichloromethane (10 mL) under an argon atmosphere and stirred overnight. The solvent volume was reduced by boiling to *ca*. 5 mL. Then, the solution was allowed to cool and placed in a refrigerator overnight. The diisopropylurea by-product was removed *via* filtration and the mother liquor was diluted with dichloromethane (25 mL), washed with 2 M H_2SO_4 (25 mL) and water (3 × 25 mL) prior to drying over $MgSO_4$ and filtration to give a brown solution. The solution was then stirred with decolorizing charcoal for 15 min and filtered through celite. The solvent was removed *in vacuo* and the material recrystallized from toluene to yield 15-crown-5 as a white powder (626 mg, 36 %). Mp. 119 °C.

^{1}**H-NMR** (500 MHz, $CDCl_3$, 295 K) δ / ppm 7.35 (d, J = 2.1 Hz, 1H, H^{A3}), 7.21 (s, 1H, H^{f}), 6.81 (m, 2H, H^{A5+A6}), 4.17 – 4.05 (m, 4H), 3.88 (m, 4H), 3.74 (m, 8H, $H^{i,j,k,l}$), 3.57 (m, 1H, H^{B3}), 3.21 – 3.14 (m, 1H, H^{B5}), 3.11 (m, 1H^{B5}), 2.45 (m, 1H, H^{B4}), 2.33 (td, J = 7.4, 2.6 Hz, 2H, H^{d}), 1.91 (td, J = 13.7, 6.9 Hz, 1H, H^{B4}), 1.82 – 1.62 (m, 4H, H^{a+c}), 1.58 - 1.43 (m, 2H, H^{b}). ^{13}**C-NMR** (126 MHz, $CDCl_3$, 295 K) δ/ppm 171.1 (C^{e}), 149.5 (C^{A2}), 145.9 (C^{A1}), 132.5 (C^{A4}), 114.9 (C^{A6}), 112.3 (C^{A5}), 107.1 (C^{A3}), 71.2 – 68.9 (8C, $C^{g,h,i,j,k,l,m,n}$), 56.5 (C^{B3}), 40.4 (C^{B4}), 38.6 (C^{B5}), 37.4 (C^{d}), 34.8 (C^{c}), 29.0 (C^{b}), 25.3 (C^{a}). EI MS m/z 471.2 [M^{+}]. Found C 56.07, H 6.90, N 3.17; $C_{22}H_{33}N_1O_6S_2$ requires C 56.03, H 7.05, N 2.97 %.

References

1. Stoop, R.L.; Wipf, M.; Müller, S.; Bedner, K.; Wright, I.A.; Martin, C.J.; Constable, E.C.; Fu, W.; Tarasov, A.; Calame, M.; *et al.* Competing surface reactions limiting the performance of ion-sensitive field-effect transistors. *Sens. Actuators B Chem.* **2015**, *220*, 500–507.
2. Willemsen, J.S.; van Hest, J.C.M.; Rutjes, F.P.J.T. Aqueous reductive amination using a dendritic metal catalyst in a dialysis bag. *Beilstein J. Org. Chem.* **2013**, *9*, 960–965.
3. Block, E.; Dikarev, E.V.; Glass, R.S.; Jin, J.; Li, B.; Li, X.; Zhang, S.Z. Synthesis, Structure, and Chemistry of New, Mixed Group 14 and 16 Heterocycles: Nucleophile-Induced Ring Contraction of Mesocyclic Dications. *J. Am. Chem. Soc.* **2006**, *128*, 14949–14961.
4. Paw, W.; Eisenberg, R. Synthesis, Characterization, and Spectroscopy of Dipyridocatecholate Complexes of Platinum. *Inorg. Chem.* **1997**, *36*, 2287–2293.
5. Wipf, M.; Stoop, R.L.; Tarasov, A.; Bedner, K.; Fu, W.; Wright, I.A.; Martin, C.J.; Constable, E.C.; Calame, M.; Schönenberger, C. Selective Sodium Sensing with Gold-Coated Silicon Nanowire Field-Effect Transistors in a Differential Setup. *ACS Nano* **2013**, *7*, 5978–5983.
